# Physical therapists' role in prevention and management of patellar tendinopathy injuries in youth, collegiate, and middle-aged indoor volleyball athletes

**DOI:** 10.1590/bjpt-rbf.2014.0126

**Published:** 2015-10-06

**Authors:** Kornelia Kulig, Lisa M. Noceti-DeWit, Stephen F. Reischl, Rob F. Landel

**Affiliations:** 1 Division of Biokinesiology and Physical Therapy, University of Southern California, Los Angeles, CA, USA

**Keywords:** physical therapy, patellar tendinopathy, volleyball

## Abstract

Patellar tendinopathy is highly prevalent in all ages and skill levels of volleyball athletes. To illustrate this, we discuss the clinical, biomechanical, and ultrasound imaging presentation and the intervention strategies of three volleyball athletes at different stages of their athletic career: youth, middle-aged, and collegiate. We present our examination strategies and interpret the data collected, including visual movement analysis and dynamics, relating these findings to the probable causes of their pain and dysfunction. Using the framework of the EdUReP concept, incorporating Education, Unloading, Reloading, and Prevention, we propose intervention strategies that target each athlete's specific issues in terms of education, rehabilitation, training, and return to sport. This framework can be generalized to manage patellar tendinopathy in other sports requiring jumping, from youth to middle age, and from recreational to elite competitive levels.

## Introduction

Volleyball became an Olympic sport in 1964 and remains one of the 10 most popular sports in the world^1^. It is estimated that elite volleyball athletes practice anywhere from 7 to 10 hours per week^2^^,^ 3 and play in matches 0.5-1.5 hours per week^2^^,^ 3. With this amount of workload, often heavily relying on jumping, it is not surprising that indoor volleyball players are susceptible to overuse injuries. One of the most frequently reported overuse injuries experienced by indoor volleyball players is patellar tendinopathy. It has been estimated that 45% of male volleyball players experience patellar tendinopathy^4^ and that male players experience symptoms more frequently than female players. Besides the workload the knee undergoes, suboptimal jumping mechanics may contribute to the development of this problem^5^.

There is a wide variety of treatment options currently available, including nonsteroidal anti-inflammatory medications^6^^-^ 8, therapeutic ultrasound^6^^-^ 8, cortisone injection^6^^-^ 8, protein-rich plasma (PRP) injections^6^^-^ 8, extracorporeal shockwave therapy^9^, eccentric exercises^8^^,^ 10 ^-^ 12, concentric exercises^11^, heavy slow-resistance training^12^, and surgical intervention^10^. At present, there is no consensus on the single optimal treatment for patellar tendinopathy in volleyball players.

The team that treats athletes with patellar tendinopathy may include athletic trainers, physical therapists, massage therapists, chiropractors, and/or acupuncturists. Management strategies may vary based on the practitioner's discipline and the athlete's age and level of competition. To illustrate the physical therapist's role in the management of age-specific and competitive level-specific patellar tendinopathy, we present the cases of a youth (Case 1: Youth), a collegiate (Case 2: Collegiate), and a middle-aged (Case 3: Middle-aged) volleyball athlete, using the EdUReP^13^concept to propose intervention strategies within the categories of Education, Unloading, Reloading, and Prevention. These intervention strategies may be utilized to manage patellar tendinopathy in other jumping sports across the age spectrum.

### Case descriptions

#### Case 1: Youth

CS (initials) is a 14-year-old high school student who is a left-handed opposite hitter for his club and school volleyball teams. He has a primary complaint of nagging left anterior knee pain. The pain began when he was 13, and the intensity has varied over the last 1.5 years. He describes the pain as a general ache with episodes of sharp pains. He is unable to determine if there are any specific aggravating factors, but does notice the aching during volleyball practice and at school. CS notes that ice and non-steroidal anti-inflammatories are helpful, but admits to using neither consistently. He reached developmental milestones in a typical timeframe and has no significant medical history.

CS currently plays/practices volleyball 3 times per week for 2 hours and competes in 2-day tournaments 1-2 times per month. He does not participate in any regular weight-lifting program. He reports that his symptoms do not limit his participation in any activity, but does notice an increase in overall intensity of symptoms at the end of a tournament.

#### Case 2: Collegiate

MM (initials) is a 19-year-old right-handed outside hitter who plays at the top competitive level of intercollegiate volleyball. One-third of the way into his second collegiate season, he reported left infrapatellar knee pain with squatting or repeated jumping. He described the pain at that time as a dull ache that intensified (from 0/10 to 6/10) toward the end of practice or a match that initially would resolve 15-20 minutes after stopping the activity. Early management included ice, non-steroidal anti-inflammatory medication, modifying weight-lifting training, using Leukotape^®^ or a patellar tendon strap, and reducing the number of jumps during practice. His symptoms improved, but returned when he resumed his "typical" resistance-training program and quantity of jumps in practice. He has had left knee pain intermittently for the last a 3 years that began at the end of his 10-month-long club season. He could "play through" his symptoms, which would resolve after not playing over the summer. He played in several beach tournaments between his first and second years and noticed that his knee would intermittently become painful if he did not adequately warm-up prior to each match. Additionally, he sustained a left grade-2 ankle sprain during the middle of his senior year of high school that prevented him from playing for 4 weeks. He reported that his ankle felt ~80% recovered upon his return to volleyball.

#### Case 3: Middle-aged

JG (initials) is a 47-year-old male engineer who has been playing for approximately 24 years. He spends 85% of his 40 to 50-hour workweek sitting down. He plays in a coed recreational indoor volleyball league 3-4 times per week and plays on the beach 1-2 times per month. He performs no other form of structured exercise.

JG reports a complex orthopedic history, beginning with a diagnosis of bilateral patellar tendinopathy 20 years ago. He was treated with only activity modification and continued playing volleyball. Eight years ago, JG underwent a left anterior cruciate ligament (ACL) reconstruction using a bone-patellar tendon-bone autograft. Despite an 8-month regimen of physical therapy, he had persistent anterior knee pain and elected to undergo a TOPAZ procedure (TOPAZ^®^ Microdebrider device, ArthroCare, Sunnyvale, CA, USA) to his patellar tendon and a lateral release, requiring an additional 8-10 months of a physical therapist's guided intervention. The TOPAZ procedure is a radiofrequency coblation applied to the pathological tissue. There is currently no reported use of the TOPAZ procedure in patellar tendon conditions, though its use in plantar heel pain and Achilles tendon pain has been reported^14^^,^ 15. To our knowledge, there are no randomized clinical trials on the efficacy of this treatment. Currently, he reports minimal left knee symptoms. Following the procedures to his left knee and return to volleyball, however, he noticed a worsening of his right patellar tendinopathy. He had no specific intervention for his symptoms until he had a TOPAZ procedure one year ago. He did not attend physical therapy following this procedure, and reports persistent knee pain that limits his ability to play volleyball at his desired level and interferes with stair-climbing and sit-to-stand/ stand-to-sit activities. His symptoms have worsened steadily over the last six weeks.

### Examination strategies

#### Movement analysis

Video of each athlete's approach jump was assessed in the sagittal and frontal planes to determine if any aberrant movements occurred during the key phases of take-off and landing that might predispose him to overload the anterior knee structures. The two key sub-phases of take-off and landing were defined as the instant of initial contact with the floor (IC) and weight acceptance (WA), during which time the athlete's presumed center of mass was lowered towards the floor. We assessed an additional sub-phase during take-off/propulsion, defined as starting from the lowest position of the presumed center of mass and ending when the feet left the ground. Critical events assessed for IC of take-off and landing included the point of contact (e.g. heel vs. whole foot vs. forefoot), the angle of ankle dorsiflexion and knee and hip flexion, and the position of the athlete's presumed center of mass relative to the point of contact (e.g. posterior center of mass relative to take-off contact point). Critical events assessed for take-off WA included the amount of hip and knee flexion and dorsiflexion (grossly equal contributions from each joint) and the direction of the center of mass relative to the point of contact (for take-off: lowering towards the ground while moving from being posterior to being directly over the point of contact; and for landing: lowering down a vertical line). Critical events for the propulsion sub-phase include grossly equal contributions in movement from the hip, knee and ankle moving the athlete's center of mass in a primarily vertical direction.

#### Objective testing

Commonly used clinical tests were performed on each athlete, including single limb balance with eyes closed, depth of single limb squat, calf strength, repetitive single limb squats, modified gluteus maximus manual muscle test, gluteus medius manual muscle test, forward plank, side plank, knee to wall ankle dorsiflexion test, hamstrings flexibility test, Thomas test, hip internal rotation, and hip external rotation. Primary objective findings for each athlete are listed in Table 1. 

**Table 1.** Relevant objective clinical findings for the youth, collegiate, and middle-aged volleyball athlete.

	**Single Leg Tests**		**Strength Testing**				**Planks**		**Flexibility**			**Hip Rotation**	
	SLS-eyes closed (sec)	SL Squat (deg)	Calf Strength (reps)	SL Squats (reps)	Gluteus Maximus (out or 5)	Gluteus Medius (out of 5)	Forward (sec)	Side (sec)	Knee-wall (cm)	Hamstring (deg)	Thomas Test (deg)	Internal Rotation (deg)	External Rotation (deg)
Youth	23/28	55/65	4/14	3/5	4-/4	3+/3+	50	24/39	12/12	46/38	-15/-5	42/42	45/45
Collegiate	4/14	55/65	23/28	6*/13	4-/4	4/5	70	34/49	12/12	48/32	-15/-20	45/45	50/50
Middle-Aged^	5/10	62/78	16/18	UA**/2	3+/4	3+/3+	51	32/40	13/12	65/55	-15/-15	30/30	35/35

First number is the measurement from the involved lower extremity; second number is from the non-involved side. Planks are bilateral, therefore no second number is needed. * with pain; UA. ** unable to perform; ^Left side=non-involved limb (both limbs initially symptomatic).

#### Diagnostic ultrasound

Gray-scale ultrasound (US) images were obtained at the distal, middle, and proximal aspects of the patellar tendon using Sonoline Antares (Siemens Medical Solutions USA Inc., Malvern, PA, USA). Longitudinal and transverse images at each tendon interval were taken by a skilled musculoskeletal ultrasonographer with 6 years of experience. The macromorphological and micromorphological characteristics of the patellar tendons were extracted from the images using standard laboratory procedures described elsewhere^16^. Color Doppler scale was used to ascertain the presence or absence of neovascularization within the tendon.

#### Biomechanical laboratory assessments

**Figure 1.**
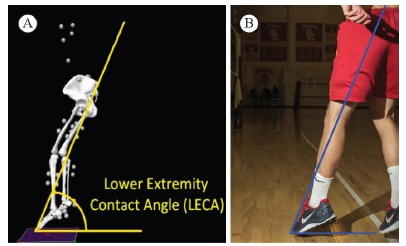
Lower extremity contact angle at the time of initial contact with the ground during landing from a jump; (A) the angle is drawn onto an image recreated from a biomechanics laboratory data collection, (B) an angle drawn onto the lower extremity posture on a photo taken on the court.

**Figure 2.**
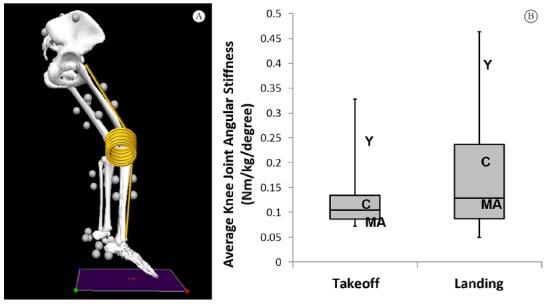
Knee Joint Torsional Stiffness: (A) Conceptual representation of knee joint torsional stiffness. The arms of the spring represent thigh and lower leg, and the coil of the spring represents the resistance provided by the muscles. The 'resistance' is represented by the computed extensor moment and the displacement by the change in joint angle; (B) Knee joint torsional stiffness (Nm/kg/degree) in a cohort of asymptomatic collegiate volleyball athletes representing the box-plot. Values for the cases presented in this case series are represented as: Y - Youth volleyball athlete, C - Collegiate volleyball athlete, and MA - middle -aged volleyball athlete.

Each athlete's spike approach jump was assessed at the Musculoskeletal Biomechanics Laboratory at the University of Southern California, Los Angeles, CA, USA. After a brief warm-up on a bicycle ergometer, each athlete was video-recorded performing a maximum of 10 volleyball jump take-offs and landings on a force plate. The data were acquired and processed using laboratory established methods described elsewhere^17^_._ The following variables were extracted from the kinematic and kinetic data:

- Lower Extremity Contact Angle (LECA) (degrees) during take-off and at landing: defined as the angle formed between the floor and a line connecting the center of pressure and the L5-S1 marker at the time of first point of contact with the ground and represents the position of the lower extremity at that point in time. The LECA, typically ranging between 61 and 78 degrees^17^ at landing, provides an estimation of the braking impulse (smaller angle = higher braking impulse) that occurs following the initial contact (Figure 1Aand 1B). The braking impulse determines the amount of force over time endured by the body. LECA at take-off is consistently smaller than at landing.

- Knee Joint Angular Stiffness (Nm/kg/degrees): joint angular (torsional) stiffness is determined by the slope of the curve formed by joint moment (kinetics) over joint displacement (kinematics). A steeper slope signifies a stiffer joint. The slope is dependent on the moment and the displacement, thus a negligible change in moment with a decrease in joint displacement will result in a higher angular joint stiffness. A conceptual comparison of the knee joint angular stiffness to a torsional spring is presented on Figure 2A 18.

### Interpretation of data

#### Case 1: Youth

The youth volleyball player has a pliable and growing neuromusculoskeletal system. Video analyses of his jumping patterns in the sagittal and frontal planes demonstrate a consistent pattern without obvious abnormalities. Objective testing showed strength and endurance deficits throughout the lower extremity and mild deficits in hamstring and hip flexor flexibility. Adult normative values were used as youth normative data are not available.

**Figure 3.**
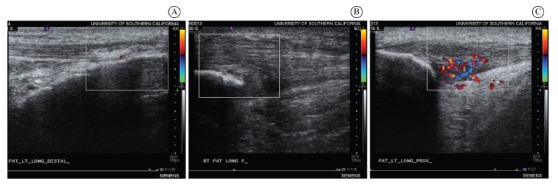
Ultrasound images of the patellar tendon: (A) distal patellar tendon of *Case 1: Youth* athlete (note brighter signal at the tibial tuberosity); (B) proximal patellar tendon in *Case 2: Collegiate* athlete (note hypoechocity and neovascularization); (C) proximal patellar tendon in *Case 3: Middle-Aged* athlete (note hypoechocity in mid-substance of the tendon, a remnant of the donor site for ACL repair).

No changes in morphology or vascularization were seen on US imaging of the patellar tendon (Figure 3A); however, there were areas of hyperechocity (brighter area) at the tibial attachment of the patellar tendon. His LECA at take-off is 60 degrees and 75 degrees at landing. Both LECA are similar to a cohort of asymptomatic male volleyball players^17^. Nevertheless, his knee joint angular stiffness is greater than the asymptomatic cohorts for both take-off and landing. 

Confirmed by US, which suggests calcific adaptation at the tibial attachment of the patellar tendon, this athlete's symptoms are likely associated with Osgood-Schlatter's Disease, resulting from mechanical overload of the patellar tendon at the tibial insertion. This is largely from overloading of bone (in this case the teno-osseous junction) due to his increased knee joint angular stiffness during take-off and landing, suggesting a stiffer weight acceptance strategy, (unpublished laboratory data) (Figure 2B) as well as limitations in strength and flexibility. 

#### Case 2: Collegiate

Of the three athletes presented, the collegiate volleyball player endures the highest volume of jumping activities, leading to a higher cumulative load. Video analysis of his approach jump at take-off and landing did not reveal any gross sagittal or frontal plane deviations. Objective testing revealed limitations in single limb balance, repetitions of single limb squatting (reproduction of pain), gluteus maximus and medius strength, forward and side plank endurance, hamstring flexibility (bilaterally), and hip flexor flexibility.

Longitudinal US images of the right knee revealed a thicker proximal patellar tendon, hypoechoic (darker) areas, and neovascularization (acquired in the Doppler mode) were present. This athlete's LECA at take-off is approximately 60 degrees, similar to the LECA of a cohort of asymptomatic male volleyball players^17^. His braking impulse, however, is lower than that cohort. When compared to symptomatic male volleyball players, his landing LECA and braking impulse are within the ranges of the asymptomatic cohort^17^. This athlete's knee angular stiffness was typical of what is seen in asymptomatic jumping athletes (unpublished laboratory data) (Figure 2B). 

Based on the results from the examinations and diagnostic US, this collegiate volleyball player is experiencing symptoms associated with patellar tendinosis (degenerative tendinopathy). The presence of hypoechocity suggests degeneration of the tendon, resulting in a more compliant tendon, while the presence of neovascularization suggests a long-standing pathological condition of the patellar tendon (Figure 3B). His LECA, tested when rested, are grossly consistent with the asymptomatic cohort, though his braking impulse at take-off is lower than the cohort, he appears to adjust for his angle at take-off by reducing the time and/or force of braking impulse. It is likely that the volume (number of repetitions and frequency) of his jumping as well as the impairments noted on the objective tests, versus alterations in his jumping mechanics, are the largest contributor to this athlete's pathology and pain pattern. 

#### Case 3: Middle-aged

The middle-aged athlete must balance life, societal roles, and his passion for volleyball. He can manipulate his repetitions and frequency of playing more easily than the athlete who still participates in organized competition. Conversely, he may have more non-volleyball related factors distracting him from being able to dedicate significant time to his training and rehabilitation program.

Video analysis of his take-off demonstrates increased knee flexion, with his hip and trunk more posterior. From the anterior view, there is a right trunk lean and the left lower extremity positioned in femoral adduction and internal rotation, while the lateral view shows that the trunk is posterior to his base of support. The anterior view of his landing strategy shows a right trunk lean and he appears to overload his right lower extremity as he absorbs the forces of landing. From the lateral view, he lands more vertically with his initial contact on his heels, which ultimately creates a jarring impact. Objective testing yielded limitations in repetitions for single limb squats (23 inch step), calf strength, single limb balance, degree of single limb squat on the right, gluteus medius and maximus strength, hamstring and hip flexor flexibility, hip internal and external rotation ROM, and forward and side plank endurance.

The proximal aspect of the patellar tendon is thicker and hypoechoic (darker) on US, though there is no neovascularization evident in the color Doppler mode. Quantitatively, the LECA at take-off is approximately 58 degrees, which is similar to a cohort of symptomatic male volleyball players^17^, and his braking impulse also lies within the range measured in the cohort of symptomatic subjects. His LECA at landing is 75 degrees which is similar to the group of asymptomatic male volleyball players^17^. The knee angular stiffness were typical of what is seen in asymptomatic jumping athletes (unpublished laboratory data) (Figure 2B). 

Based on the collective results from movement analysis, objective testing, diagnostic ultrasonography and biomechanical assessments, this athlete has signs and symptoms consistent with patellar tendinosis. Similar to Case 2: Collegiate, this athlete's US images indicate tendon degeneration (hypoechocity), a more compliant tendon, and the lack of neovascularization suggests an absence of reparative process within the tendon (Figure 3C). This is likely most related to his take-off and landing mechanics, which place an overload on the patellar tendon. 

### Intervention plan

There is a large range of options in managing an athlete with patellar tendinopathy. The choice of options is dependent upon multiple factors, including position played, severity/irritability of symptoms, duration of competitive season, chronicity of problem, amount of time left in season, current weight-training regimen (if any), as well as age. Considering these factors is critical to determining the appropriate management of an athlete with patellar tendinopathy. While pain alleviating interventions (including anti-inflammatories, electrical stimulation, US, phonophoresis, iontophoresis, Platelet Rich Plasma (PRP) or cortisone injections, extracorporeal shockwave therapy and ice) are appropriate and helpful in the short term, it is a disservice to the athlete if the factors contributing to the development of patellar tendinopathy are not addressed.

The EdUReP^13^ concept is useful in the management of individuals with tendinopathy, including athletes^19^. The concept stresses Education, Unloading, Reloading, and Prevention^13^and has been primarily used in the management of individuals with posterior tibial tendinopathy^20^^-^ 22.

- Education: Education regarding the disease process, pathophysiological changes in the tendon, the intervention plan, and the cyclical nature of tendinopathy is essential. It is especially important that the athlete understand that pain is not synonymous with damage within the tendon, but is a typical reaction in a tendinopathic tendon. Discussing aberrant movement patterns and how to alter them is another key point.
- Unloading: A period of unloading the tendon is necessary, though the duration of unloading may vary due to a multitude of reasons. Clinical experience suggests 2-4 weeks of unloading is optimal, though it is unclear on what time frame is ideal in athletes during their competitive season. As always, there will be a conflict between appropriate unloading and the pressures of returning to competition, and an appropriate balance must be found. Unloading may include direct or indirect forms of modifying activity. Examples of direct forms include: bracing or taping variations. Examples of indirect forms include modifying weight-lifting activities, modifying quantity of jumps, optimizing squatting mechanics, and mechanics during the take-off and landing.
- Reloading: The concept of reloading applies to the patellar tendon itself and addressing strength impairments via resistive exercises in remote regions. There is inconsistent information regarding the preferable way to reload the patellar tendon, but the most consistently utilized is eccentric loading. Visnes et al.^23^ suggests a slow decline squat on the involved lower extremity, using hands and/or the other limb to return to full upright position. Three sets of 15 repetitions should be performed twice daily, beginning on flat land and then progressing to a 25-degree angle wedge. Knee flexion to a minimum of 60 degrees should be emphasized, if pain allows (<5/10 overall), and preferably be an eccentric motion only (Figure 4).
  **Figure 4.**
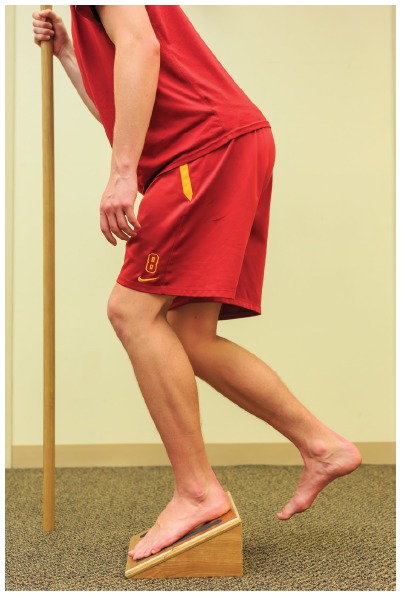
Slow repeated single limb squat lowering exercise on a decline board providing for eccentric loading to the patellar tendon. This is tissue-specific re-loading.
- Prevent: The prevention aspect of the EdUReP concept primarily consists of strategies that address factors contributing to patellar tendon overload. As the previous case studies suggest, this might include appropriate pre-season training (perhaps including eccentric training), improving jump/landing mechanics, and proper adjustment of training volume (whether jumps or resistance training).

### Specific interventions for each case

#### Case 1: Youth

The athlete and his parents were educated regarding the pathophysiology of Osgood-Schlatter's. They were informed that this pathology is likely a result of mechanical overload at the tibial tubercle, that it is a self-limiting disorder that has a good prognosis, and that pain does not correlate with structural damage. The intervention plan addressed areas that contributed to, but were not specifically at, the painful location (e.g. strength and flexibility of the hip and ankle).

Specific unloading techniques included fat pad unloading during practice and matches. Modifications to strength training were unnecessary, as he was not currently involved in a weight training program; however, repetitive jumping drills (i.e. multiple repetitions of hitting) were eliminated from practice for 3-4 weeks.

**Figure 5.**
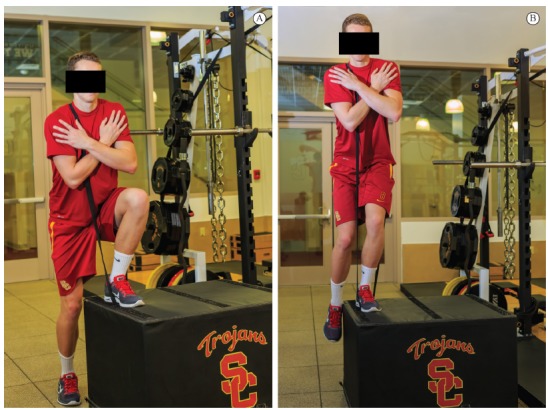
Resistive step-up exercises targeting the knee extensors in an upright position, requiring the control of balance. This is non-tissue specific re-loading. A: Left; B: Right.

Specific isolated reloading of the patellar tendon is not indicated in this athlete as the pathology is not located within the tendon, and eccentric loading would likely aggravate the condition. However, reloading of other body regions is indicated. Exercises for the quadriceps included lateral step ups with a band to increases resistance (Figure 5), forward lunges, forward step ups and step downs, and side lunges with ipsilateral lateral reach. Prevention strategies included active and passive static and dynamic multiplanar hamstring and quadriceps stretches. Additional exercises included double limb (progressing to single limb) bridges, sidelying clams, single limb heel raises, forward step-up with posterior lunge, balance and reach, forward planks, and hurdle jumps. 

The therapist discussed the biomechanical laboratory data with the athlete, coach, and athletic trainer. The knee angular joint stiffness was explained using the illustration of a torsional spring in Figure 2Aand its representation of concurrent change in knee net joint moment and change in knee angular displacement. For example, a quieter landing strategy would represent similar net joint moment but larger joint displacement. This concept was then implemented during practice by asking the athlete to pay attention to the sound accompanying the jumps, namely "louder" for a stiffer landing and "quieter" for less stiff (softer) landing, the second being desirable. 

#### Case 2: Collegiate

The collegiate outside hitter's future promises a lot of jump repetitions, both in the remaining season and his collegiate career. It is important that he understands that his symptoms are a result of tendinopathic changes and that there is a minimal inflammatory response present. He must understand that the tendon has attempted to heal but failed to do so, leaving him with a chronic problem. However, pain is not an indication of acute tissue damage and thus he can work through some level of pain. More importantly, while his symptoms will likely improve with time a significant lessening of pain may not occur during the competitive season; this will largely depend on his ability to control the quantity of jumps and his strength and conditioning program^23^. In addition, the problem may reoccur, which may be a source of frustration for him.

Since he continued to participate at nearly the same level, with only a slight reduction in the number of jumps during practice, he was instructed to use a patellar tendon strap during weight lifting and used Leukotape^®^to unload the tendon during practice and games. His strength and conditioning program was modified to decrease the quantity of quadriceps dominant exercises, increase the quantity of gluteal strengthening, and alter the squatting pattern to bias a hip dominant (hip hinge) pattern versus a quadriceps dominant (knee forward) pattern. Specific reloading was accomplished using eccentric loading as described previously.

**Figure 6.**
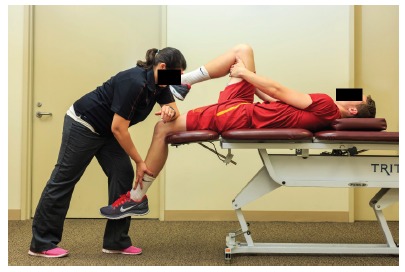
A passive soft-tissue technique to the distal quadriceps region aiming at improving tissue extensibility.

Interventions to decrease the frequency of recurrences were initiated. Improvement in hamstring flexibility, extensibility of the distal quadriceps (Figure 6), hip extension range of motion, and strength of the hip extensors and abductors were addressed during the season and a maintenance program was initiated for the off-season. Plyometric activities were minimized during the season, since the primary concern was reducing the number and frequency of jumps and not improvement of his jumping mechanics. 

#### Case 3: Middle-aged

This athlete had been dealing with patellar tendinopathy for several years. Education played a key role in his rehabilitation. He learned about the pathophysiologic changes evident in his diagnostic US, the changes that occur in the musculoskeletal system with age, the link between his movement patterns and the overload they created on the patellar tendon, and that his prognosis depended on his ability to be "fit for playing" versus using volleyball for fitness. For the interventions to be most beneficial, he needed to understand that specific interventions to the patellar tendon, as well as other regions, were required. The discussion regarding altering his landing mechanics should commence early but with the understanding that significant changes will take time to accomplish. The importance of regular exercise and cardiovascular fitness as a preventative measure is a necessary discussion. It is imperative that he understand that, due to age-related changes in his physiology, it is best to maintain a basic level of flexibility and strength. One of the most important things to address is the time element - finding a concise workout regimen that will be easily adhered to by the middle-aged athlete.

Specific patellar tendon unloading was essential for this athlete, accomplished directly using a patellar tendon strap or Leukotape^®^. Indirect tendon unloading included a stretching program addressing hamstrings, quadriceps, and the calf. Providing an adequate rest period was easier in this athlete because he played at a recreational level, and in total, he took approximately 3 weeks off from volleyball. When the athlete had a significant reduction in symptoms, the athlete began an eccentric patellar tendon loading program as described previously. The athlete began with his foot flat on the ground and progressed to a decline board of at least 25 degrees to decrease the calf contribution during the eccentric (lowering) phase of the squat, increase the load on the patellar tendon, and to stimulate the tenocytes to change the collagen fibers and alter the mechanical properties of the patellar tendon^24^. He was instructed to perform this exercise twice daily, 3 sets of 15 repetitions (Figure 4). The prevention portion of the EdUReP concept may be the most influential in decreasing the frequency of recurrence of patellar tendinopathy in this athlete. Manual, static, and dynamic stretching of the quadriceps, hamstrings, and calf is important to help counteract the prolonged positioning of this athlete during his workday. Specific instructions to perform the stretches correctly are imperative to avoid accidently overloading another body region. Additionally, it is best to choose stretches that can be performed easily at home, work, or at the court/beach. Strengthening intervention targeted multiple areas, including trunk stabilization and endurance, hip, ankle, and knee deficits. Though tedious, it is likely ideal to begin a strengthening program that isolates the muscles to ensure adequate recruitment and minimal compensations prior to transitioning into larger, more dynamic movements. Examples of isolated strengthening include sidelying clams and bridging (double-single limb-exercise ball), crunches, and single limb heel raises. These exercises can progress to double-single limb squatting with various height arm reaches and to various sides, multidirectional lunging with and without arm movements, forward and side planks, and balance/agility activities. During the instructional period, proper form is important, and he must understand that pain is expected during the activity. Plyometric activities should be initiated only after pain levels have reduced and more optimal neuromuscular control during dynamic movements are obtained. There are many plyometric activities to choose from; however, appropriate dosage and progression of the quantity of jumps is as important as his take-off and landing mechanics. A gradual transition into a full approach jump is an excellent training tool. 

Fitting in the conceptual framework "to Educate", the therapist and the athlete reviewed the videos of his take-off and landing and discussed the relevance of body position at the moment of contact with the ground (LECA) and the subsequent interaction with the ground, focusing on the degree of knee flexion (knee joint torsional stiffness). The optimal strategies were discussed in light of jump performance. Technique cues were suggested, such as "bring your legs underneath you" in preparation for landing to avoid a smaller LECA and therefore lesser braking impulse. Once in the ground contact phase, optimal rate and amount of knee joint flexion were discussed in light of laboratory data on knee joint torsion stiffness. These concepts were consequently explored during practice.

### Summary of intervention

The management of patellar tendinopathy in volleyball players is more complex than the etiology might suggest. The presence of macro- and micromorphological changes in the tendon, the athletes' age, and their competitive level are only a few of the factors that need to be considered when determining the optimal management of this condition.

The EdUReP concept helps determine a treatment plan for each of these athletes, though not all aspects of the concept are relevant to every case. Education of the pathology and treatment plan was essential for each athlete presented, as was a period of unloading. However, the manner of unloading varied for each athlete. It was important and necessary to address strength and/or flexibility deficits for all three athletes, though only those with tendinopathic changes on diagnostic ultrasound (the middle-aged and collegiate players) benefitted from an eccentric training program. Each athlete went through a period of reloading, though the type of exercise varied. Specific exercises targeting prevention of further injury or re-injury varied for each athlete, though largely included the broad topics of flexibility, strengthening, and neuromuscular control. For certain athletes, it was important to discuss and alter jumping patterns and changes in lifestyle habits.

A brief synopsis of the similarities and differences in treatments are presented in Table 2. 

**Table 2:** Management synopsis of the youth, collegiate, and middle-aged volleyball athlete.

	CASE 1: YOUTH	CASE 2: COLLEGIATE	CASE 3: MIDDLE-AGED
	*The following aspects of the education portion were discussed with the athlete*	*The following aspects of the education portion were discussed with the athlete*	*The following aspects of the education portion were discussed with the athlete*
**Education**	• Condition of the tendon bone interface as the pathology which should recover	• Condition is not an inflammatory process	• Condition is not an inflammatory process
		• Periods of exacerbation	• Periods of exacerbation
		• Demand of the position played	• Need to change fitness level
		• Time of the season, may not be able to rest as much	• Volleyball not making him fit, must be "fit to play"
		• May not improve during season	• Alteration of play and practice to allow rest
**Un-Loading**			• Change of take-off and landing patterns
		• Alteration of play and practice	• Change the level of play with more rest
	• Tape, patellar strap to change stress at the symptom region	• Alteration of weight training regimen	• Flexibility exercises to reduce stress on tendon
	• Reduce practice and competition when symptoms are elevated	• Use of tape or strap at the patellar tendon	• Possible use of strap or tape
**Re-Loading**			• Eccentric loading of patellar tendon, 3 x15 twice per day
	• Due to lack of tendon pathology, the eccentric program is not part of the Youth's intervention program	Eccentric loading of patellar tendon, using 3 x15 twice per day working towards use of decline board for 12 weeks	• Long term eccentric use 2-3 times a week
**Prevention**		• Athlete to compete through long season without loss of performance	• Alteration of fitness program, with inclusion of comprehensive lower extremity flexibility program
	• Long term flexibility program to follow up manual therapy intervention	• Additional stretching program for LE of hamstrings, distal quad and long hip flexors	• Strength training for trunk and lower extremity to reduce demand on quadriceps and patellar tendon
	• Strength training for trunk, hip abduction, hip extension, calf	• Specific strength training of hip extensors and abductors, first in isolation and progressed	• Progress from more isolated muscle activation to patterns of movement and change towards plyometrics
	• Neuromuscular and Movement Reeducation to plyometrics	• In-season jumping activities decreased in practice and in weight training	• Sustaining the change of fitness should ensure continued ability to perform
